# Supplementary material for: Design and characterization of HIV-1 vaccine candidates to elicit antibodies targeting multiple epitopes
Source: J Exp Med. 2025 Aug 12;222(10):e20250693. doi: 10.1084/jem.20250693 (PMC12341506; doi:10.1084/jem.20250693)
Supplement: Table S1 — shows EM data collection and processing statistics. [file jem_20250693_tables1.docx]

**Table S1. EM data collection and processing statistics**

**3nv.2 SOSIP**

| **Data collection conditions** |  |
| --- | --- |
| Microscope | Talos Arctica |
| Voltage (kV) | 200 |
| Camera | Gatan K3 |
| Magnification | 45,000x |
| Frames per movie | 40 |
| Recording mode | counting |
| Dose rate (e-/pixel/s) | 21.234 |
| Total electron dose (e-/Å2) | 45 |
| Defocus range (μm) | -0.8 to -1 |
| Pixel size (Å) | 0.435 (super-resolution) |
| Micrographs collected | 2,100 |
| Total extracted particles | 142,241 |
|  |  |
| **EMD** |  |
| Particles in class | 24,310 |
| Symmetry | C3 |
| Map resolution (Å) | 6.6 |
| FSC threshold | 0.143 |
